# Toxicity of Metals to a Freshwater Ostracod: *Stenocypris major*

**DOI:** 10.1155/2011/136104

**Published:** 2011-04-10

**Authors:** Mohammad Shuhaimi-Othman, Nadzifah Yakub, Nur-Amalina Ramle, Ahmad Abas

**Affiliations:** School of Environmental and Natural Resource Sciences, Faculty of Science and Technology, National University of Malaysia (UKM), 43600 Bangi, Selangor, Malaysia

## Abstract

Adults of freshwater ostracod *Stenocypris major* (Crustacea, Candonidae) were exposed for a four-day period in laboratory conditions to a range of copper (Cu), cadmium (Cd), zinc (Zn), lead (Pb), nickel (Ni), iron (Fe), aluminium (Al), and manganese (Mn) concentrations. Mortality was assessed, and median lethal times (LT_50_) and concentrations (LC_50_) were calculated. LT_50_ and LC_50_ increased with the decrease in mean exposure concentrations and times, respectively, for all metals. LC_50_s for 96 hours for Cu, Cd, Zn, Pb, Ni, Fe, Al, and Mn were 25.2, 13.1, 1189.8, 526.2, 19743.7, 278.9, 3101.9, and 510.2 *μ*g/L, respectively. Metals bioconcentration in *S. major* increases with exposure to increasing concentrations, and Cd was the most toxic to *S. major*, followed by Cu, Fe, Mn, Pb, Zn, Al, and Ni (Cd>Cu>Fe>Mn>Pb>Zn>Al>Ni). Comparison of LC_50_ values for metals for this species with those for other freshwater crustacean reveals that *S. major* is equally or more sensitive to metals than most other tested crustacean.

## 1. Introduction

Widespread uses of metals, the legacies of past contamination and new technologies, continue to pose and important ecological risk in aquatic environment [1]. Metals such as Cu, Cd, Zn, and Pb are released from natural sources as well as human activity. Impact of these metals to the environment is an increasing problem worldwide. Malaysia, as a developed country, is no exception and faces metals pollution caused especially by anthropogenic activities such as manufacturing, agriculture, sewage, and motor vehicle emissions [2, 3]. Metals research in Malaysia, especially using organisms as bioindicator, is still scarce. Therefore, it is important to conduct studies with local organisms that can be used to gain data on metal toxicity, to determine the organism's sensitivity and to derive a permissible limit for Malaysian's water that can protect aquatic communities. Managing trace metal contamination requires understanding the concentration dependence of toxicity. Toxicity testing is an essential tool for assessing the effect and fate of toxicants in aquatic ecosystems and has been widely used as a tool to identify suitable organisms as a bioindicator and to derive water quality standards for chemicals. Toxicity testing provides the underpinning for traditional regulatory approaches for all chemicals and is an important part of many risk assessment [1, 4]. 

Ostracods are microscopic, bivalve crustaceans, with valve of low-Mg calcite. Ostracods are ubiquitous in fresh waters. They are mainly benthic, and fairly common in shallow water bodies. On area basis, small lentic systems such as pond and pools support more ostracods taxa than large lakes. They also thrive in temporary habitats including rice fields and containers like tins, discarded tires, tree holes, crab hole, and so forth. Benthic ostracods are mainly detritivores and also readily feed on dead animals. Ostracods form an important component in the food chain of some fish. Freshwater ostracods reproduce sexually and also by parthenogenesis. Females are more abundant and common than males. They have resistant eggs that can withstand adverse environmental conditions. Ostracods can also aestivate (dormant) or hibernate as resistant larval stages. Like other crustacean, ostracods moult, generally passing eight stages to reach adulthood, and life cycle may last a few months or more than 2 years. The most prevalent genus in Southeast Asia is *Strandesia* with about 30 species. Other genera that are common occurrence are *Stenocypris*, *Hemicypris,* and *Cypretta* [5, 6]. There are approximately 11 species of *Stenocypris* genus.* Stenocypris major* is widely distributed and has been reported from South America, Africa, and Asia. It is a nektobenthic species and is known to be eurytopic species, that is, taxa with a broad tolerance for ecological conditions and it prefers shallow and running water [7]. 

Ostracods have been used as one of freshwater invertebrates in ecotoxicological studies and as a test model organisms for environmental, paleoenvironmental, and toxic stress studies and also for toxicity monitoring of soil and river sediment [8–10]. Ruiz et al. [11] suggest that ostracods are highly sensitive to heavy metal pollution, oil-discharges, and anoxic conditions, and a study by Khangarot and Das [10] demonstrated the need to include crustacean ostracods in a battery of biotest to detect the presence of hazardous chemicals in soils, sewage sludge, sediments, and aquatic systems. Some metals toxicity studies have been conducted with freshwater ostracods such as *Cypris subglobosa* [10, 12], and toxicity to organic pollutants with *Ilyocypris dentifera*, *Cypridopsis vidua,* and *Cypretta seurati* [13]. However, no toxicity studies have been reported on *S. major* in the literature especially to metals. The purpose of this study was to determine the acute toxicity of Cu, Cd, Zn, Pb, Ni, Fe, Al, and Mn to freshwater ostracod, *S. major,* and to examine bioconcentration of these metals in the body after four days of exposure. 

## 2. Materials and Methods

Ostracods were collected from filter system of fish pond in Bangi, Selangor, Malaysia. The filter system was consisting of several layers of filter mate and made from polyester wool and the water is continuously circulating using water pump from the fish pond to the filter, and back to the pond. Identification of species was based on Victor and Fernando [5] and Victor [6]. Prior to toxicity testing, the ostracods were acclimatized for one week under laboratory conditions (28–30°C with 12 h light : 12 h darkness) in 50-L stocking tanks using dechlorinated tap water (filtered by several layers of sand and activated carbon; T.C. Sediment Filter) aerated through an air stone. During acclimation, the ostracods were fed with commercial fish food Aquadene. The standard stock solution (100 mg/L) of Cu, Cd, Zn, Pb, Ni, Fe, Al, and Mn were prepared from CuSO_4_·5H_2_O, CdCl_2_·2.5H_2_O, ZnSO_4_·7H_2_O, Pb(NO_3_)^2^, NiSO_4_·6H_2_O, FeCl_3_, Al_2_(SO_4_)_3_·18H_2_O, and MnSO_4_·H_2_O, respectively. The stock solutions were prepared with deionized water in 1-L volumetric flasks. Acute Cu, Cd, Zn, Pb, Ni, Fe, Al, and Mn toxicity experiments were performed for a four-day period using adult ostracods (approximately 1.5 mm body length, mean wet weight 0.3 mg) obtained from stocking tanks. Following a range finding test, five Cu (32, 56, 100, 560, and 870 *μ*g/L), Cd (56, 87, 320, 560, and 870 *μ*g/L), Zn (560, 1000, 2400, 3200, and 5600 *μ*g/L), Pb (560, 1000, 3200, 5600, and 10000 *μ*g/L), Ni (1800, 3200, 5600, 8700, and 10000 *μ*g/L), Fe (560, 750, 1000, 3200, and 5600 *μ*g/L), Al (1000, 5600, 8700, 10000, and 18000 *μ*g/L), and Mn (560, 870, 1000, 3200, and 5600 *μ*g/L) concentrations were chosen. Metal solutions were prepared by dilution of a stock solution with dechlorinated tap water. A control with dechlorinated tap water only was also used. The tests were carried out under static conditions with renewal of the solution every two days. Control and metal-treated groups each consisted of five replicates of four randomly allocated ostracods in a 10 mL glass vial containing 8 mL of the appropriate solution. No stress was observed for the ostracods in the solution, indicated by 100% survival for the ostracods in the control water until the end of the study. A total of 20 animals per treatment/concentration were used in the experiment and a total of 820 animals were employed in the investigation [14, 15]. Samples of water for metal analysis taken before and immediately after each solution renewal were acidified to 1% with ARISTAR nitric acid (65%) before metal analysis by flame or furnace Atomic Absorption Spectrophotometer (AAS–Perkin Elmer model AAnalyst800) depending on the concentrations. 

During the toxicity test, the ostracods were not fed. The experiments were performed at room temperature of 28–30°C with photoperiod 12 h light : 12 h darkness, using fluorescent lights (334–376 lux). Water quality parameters (pH, conductivity, and dissolved oxygen) were measured every two days using portable meters (model Hydrolab Quanta), and water hardness samples (0.45 *μ*m filtered) were fixed with ARISTAR nitric acid and measured by flame atomic absorption spectrophotometer. Mortality was recorded every 3 to 4 hours for the first two days and then at 12 to 24 hour intervals throughout the rest of the test period. The criteria used to determine mortality were failure to respond to gentle physical stimulation. Any dead animals were removed immediately.

At the end of day four, the live ostracods were used to determine bioconcentration of the metals in whole body according to the concentrations used. The ostracods were rinsed with distilled water and each sample contained three replicates of three to five animals in a glass test tube (depending on how many live animals were left) and was oven dried (80°C) for at least 48 hours before being weighed. Each replicate was digested (whole organism) in 1.0 mL Aristar nitric acid (65%) in a block thermostat (80°C) for 2 hours. Upon cooling, 0.8 mL of hydrogen peroxide (30%) was added to the solutions. The test tubes were put back on the block thermostat for another 1 hour until the solutions became clear. The solutions were then made up to 25 mL with addition of deionized water in 25-mL volumetric flasks. Efficiency of the digestion method was evaluated using mussel and lobster tissue reference material (SRM 2976 and TORT-2, National Institute of Standard and Technology, Gaithersburg, USA and National Research Council Canada, Ottawa, Ontario, Canada, resp.). Efficiencies obtained were within 10% of the reference values. To avoid possible contamination, all glassware and equipment used were acid-washed (20% HNO_3_), and the accuracy of the analysis was checked against blanks. Procedural blanks and quality control samples made from standard solutions for Cu, Cd, Zn, Pb, Ni, Fe, Al, and Mn were analyzed in every ten samples in order to check for sample accuracy. Percentage recoveries for metals analyses were between 85–105%.

Median lethal times (LT_50_) and concentrations (LC_50_) for the ostracods exposed to metals were calculated using measured metal concentrations. FORTRAN programs based on the methods of Litchfield [16] and Litchfield and Wilcoxon [17] were used to compute and compare the LT_50_ and LC_50_. Concentration factors (CFs) were calculated for whole animals as the ratio of the metals concentrations in the tissues to the metals concentration measured in the water. 

## 3. Results and Discussion

In all data analyses, the actual, rather than nominal, Cu, Cd, Zn, Pb, Ni, Fe, Al, and Mn concentrations were used (Table 1). The mean water quality parameters measured during the test were pH 6.51 ± 0.01, conductivity 244.3 ± 0.6 *μ*S/cm, dissolved oxygen 6.25 ± 0.06 mg/L, and total hardness (Mg^2+^ and Ca^2+^) 15.63 ± 2.74 mg/L as CaCO_3_. 

**Figure 1:**
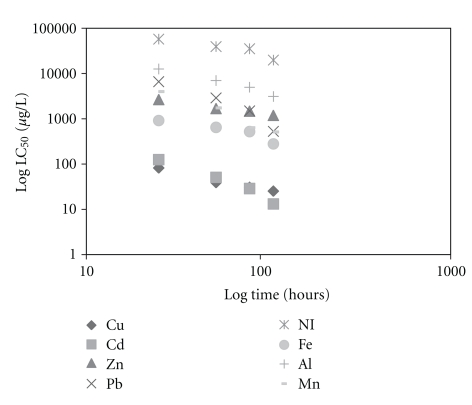
The relationship between median lethal concentration (LC_50_) and exposure times for *S. major*.

**Figure 2:**
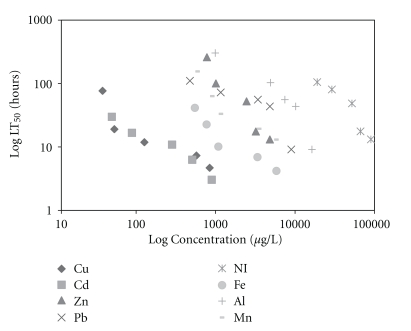
The relationship between median lethal time (LT_50_) and exposure concentrations for *S. major*.

One hundred percent of control animals maintained in dechlorinated water survived throughout the experiment. The median lethal times (LT_50_) and concentrations (LC_50_) increased with a decrease in mean exposure concentrations and times, respectively, for all metals (Tables 1 and 2). However, the lethal threshold concentration could not be determined since the toxicity curves (Figures 1 and 2) did not become asymptotic to the time axis within the test period. Figures 1 and 2 also show that Cd was the most toxic to *S. major*, followed by Cu, Fe, Mn, Pb, Zn, Al, and Ni. Similar results were reported for the ostracod *Cypris subglobosa* [10]. Arambašić et al. [18] found that with *Daphnia magna*, the order of toxicity was Cu>Zn>Pb, and Bacher and O'Brien [19] showed that Cu was more toxic than Pb to *Daphnia carinata*. 

This study showed that LC_50_s for 96 hours for Cu, Cd, Zn, Pb, Ni, Fe, Al, and Mn were 25.2, 13.1, 1189.8, 526.2, 19743.7, 278.9, 3101.9, and 510.2 *μ*g/L, respectively (Table 2). Few studies were reported on the toxicity of metals to ostracods. Khangarot and Ray [12] showed that toxicity of Cu to ostracod *Cypris subglobosa* increases as pH of the test medium decreases from 8.5 (EC_50_ = 5.1 mg/L) to 5.5 (EC_50_ = 0.35 mg/L) and vice versa. Khangarot and Das [10] showed that the 48 h EC_50_s (immobilization) for Cu, Cd, Zn, Pb, Ni, Fe, Al, and Mn for *Cypris subglobosa* were 0.55, 0.82, 85.04, 40.19, 75.78, 115.2, 100.90, and 11.77 mg/L, respectively. The toxicity baseline database on ostracod is still deficient; therefore, a comparison of LC_50_ values with other freshwater crustacean especially cladocerans, amphipods, and few ostracods is shown in Table 3. This study showed that for all metals tested, *S. major* showed highest sensitivity compared to other species such as ostracod *Cypris subglobosa*, cladoceran *Daphnia carinata,* and amphipod *Hyalella azteca*, except for Pb and Ni (Table 3). Present study showed that for Cu, Cd, Zn, Fe, Al, and Mn, 96h-LC_50_ values obtained were lower than for other ostracod, cladocerans, and amphipods (Table 3). This indicated that *S. major* is equally or more sensitive than most of the reported species for metals. Von Der Ohe and Liess [29] showed that 13 taxa belonging to Crustacea were among the most sensitive to metal compounds and concluded that taxa belonging to Crustacea are similar to one another and to *Daphnia magna* in terms of sensitivity to organics and metals. 

In comparison with other freshwater ostracod (*Cypris subglobosa*) (Table 3), this study showed that LC_50_s for *S. major* were lower compared to EC_50_ (immobilization) of *C. subglobosa* for all the metals tested although our end point of study was higher (mortality) compared to *C. subglobosa* (immobilization) [10]. These differences are probably due to different species used, age, size of the organism, test methods, and water quality such as water hardness, as this can affect toxicity [30, 31]. In the present study, water hardness was considered low, and the water was categorized as soft water (<75 mg/L as CaCO_3_) compared to study by Khangarot and Das [10] where they used hard water (245 mg/L as CaCO_3_). 

**Figure 3:**
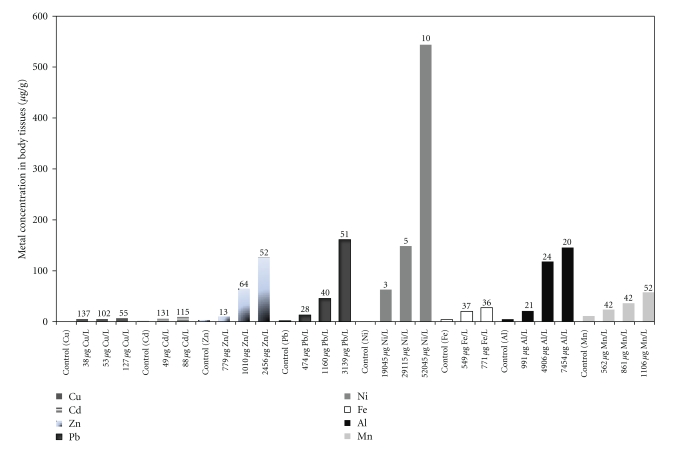
Bioconcentration of Cu, Cd, Zn, Pb, Ni, Fe, Al, and Mn (mean) in *S. major* after a four-day exposure to different concentrations of Cu, Cd, Zn, Pb, Ni, Fe, Al, and Mn. Concentration factor (CF) is indicated at the top of each bar.

Bioconcentration of Cu, Cd, Zn, Pb, Ni, Fe, Al and Mn in surviving *S. major* are as shown in Figure 3. Bioconcentration data for live ostracods were obtained from three Cu (38, 53, and 127 *μ*g/L), Zn (779, 1010, and 2456 *μ*g/L), Pb (474, 1160, and 3139 *μ*g/L), Ni (19045, 29115, and 52045 *μ*g/L), Al (991, 4906, and 7454 *μ*g/L) and Mn (562, 861, and 1106 *μ*g/L) concentration exposures, and two Cd (49 and 88 *μ*g/L) and Fe (549 and 771 *μ*g/L) concentration exposures. In general, Cu, Cd, Pb, Zn, Ni, Fe, Al, and Mn bioconcentration in *S. major* increases with increasing concentration exposure. Luoma and Rainbow [1] reported that the uptake of trace metals from solution by an aquatic organism is primarily concentration dependent. The higher the dissolved concentration of the trace metal is, the higher will be the uptake of the metal from solution into the organism, until the uptake mechanism becomes saturated. Concentration factor (CFs) also showed some trend of increases with increasing concentration exposure (Figure 3) especially for Pb, Ni, and Mn. In general, the highest CF was noted for Cu (137) and Cd (131), and the lowest CF for Ni (3). Similar results were reported by Timmermans et al. [32] with *Chironomus riparius*, which showed that among four metals (Cu, Cd, Zn, and Pb), Cd had the highest bioconcentration factor (BCF). Shuhaimi-Othman and Pascoe [33] also showed that Cd had the highest BCF followed by Cu and Zn with amphipod *Hyalella azteca*.

 Higher uptake of Cd in invertebrates probably due to availability of the uptake route through “major ion” channels as Cd can enter into the cell through Ca channels as both metals have very similar ionic radius [34]. Higher accumulation of Cd in *S. major* also seems to be associated with net accumulation of these metals. A study by Vijayram and Geraldine [35] with freshwater prawn, *Macrobrachium malcolmsonii*, showed that the prawn accumulated the nonessential metal Cd at all exposure levels (6.3–157 *μ*g/L) without any regulation. However, the prawn regulated the essential metal Zn until it reached threshold level (373 *μ*g/L), at which regulation collapsed and net accumulation began. Borgmann et al. [36] showed that the amphipod *Hyalella azteca* was capable of regulating Cu but unable to regulate Zn as effectively and did not regulate Hg, Cd, and Pb. Krantzberg and Stokes [37] reported that chironomid larvae were able to regulate or control the accumulation of Ni and Zn but could not regulate Pb and Cd. Comparison of uptake rate in aquatic organisms showed that in general the order of the uptake rate constant is Ag>Zn>Cd>Cu>Co>Cr>Se [1]. This discrepancy is probably due to short time of exposure (four days) to metals in this study. 

## 4. Conclusions

This study showed that *S. major *was equally or more sensitive to metals compared to other freshwater crustaceans. Cd was the most toxic to *S. major* followed by Cu, Fe, Mn, Pb, Zn, Al, and Ni. A comparison of bioconcentration of metals in *S. major* showed that among the eight metals studied, Cu and Cd were the most accumulated and Ni was the least accumulated. This study indicates that *S. major* is a potential bioindicator organism of metals pollution and in toxicity testing. 

## Figures and Tables

**Table:** (a)

Measured Cu concentration (*μ*g/L)	LT_50_ (h)	95% Confidence limits	Measured Cd concentration (*μ*g/L)	LT_50_ (h)	95% Confidence limits	Measured Zn concentration (*μ*g/L)	LT_50_ (h)	95% Confidence limits
37.78	76.69	27.15–216.57	49.41	29.89	14.44–61.9	779	260.33	743.38–1562.33
53.18	19.05	9.02–40.26	88.57	16.72	8.53–32.77	1010	100.63	63.30–159.99
126.96	11.86	6.06–23.24	282.91	10.87	5.87–20.12	2456	52.52	32.18–85.71
573.91	7.31	4.30–12.43	508.43	6.29	3.77–10.48	3213	17.59	11.88–26.05
842.65	4.68	2.82–7.77	893.52	3.04	2.12–4.28	4822	13.17	10.31–16.82

**Table:** (b)

Measured Pb concentration (*μ*g/L)	LT_50_ (h)	95% Confidence limits	Measured Ni concentration (*μ*g/L)	LT_50_ (h)	95% Confidence limits	Measured Fe concentration (*μ*g/L)	LT_50_ (h)	95% Confidence limits
475	110.26	79.32–153.28	19045	105.13	71.40–154.79	548.93	41.36	18.84–90.81
1160	72.39	53.61–97.76	29115	80.76	58.88–110.77	771.18	22.66	11.64–44.10
3410	55.66	42.32–73.21	52045	48.61	32.46–72.78	1084.0	10.13	5.53–18.57
4829	43.55	29.86–63.51	66665	17.59	11.88–26.05	3370.02	6.93	4.02–11.93
8973	9.15	5.90–14.17	90290	13.17	10.31–16.82	5836.64	4.20	2.82–6.25

**Table:** (c)

Measured Al concentration (*μ*g/L)	LT_50_ (h)	95% Confidence limits	Measured Mn concentration (*μ*g/L)	LT_50_ (h)	95% Confidence limits
991	303.15	59.21–1552.17	562.28	155.41	53.84–448.63
4907	103.22	57.48–185.34	861.37	63.31	24.24–165.33
7454	55.66	42.32–73.21	1106.36	33.23	13.65–80.88
10210	43.55	29.86–63.51	3351.91	19.36	9.51–39.41
16348	9.15	5.90–14.17	5519.08	13.08	6.66–25.71

**Table:** (a)

Time (hour)	LC_50 _ (*μ*g/L) for Cu	95% Confidence limits	LC_50 _ (*μ*g/L) for Cd	95% Confidence limits	LC_50 _ (*μ*g/L) for Zn	95% Confidence limits	LC_50 _ (*μ*g/L) for Pb	95% Confidence limits
24	82.17	39.02–141.5	125.18	77.69–180.97	2655.27	2148.19–3282.04	6582.57	4920.11–8806.77
48	38.84	19.3–55.82	50.73	21.7–78.3	1682.70	1365.77–2073.17	2885.86	7919.06–4339.71
72	30.67	10.07–42.46	28.76	0.49–47.82	1475.74	1204.16–1808.58	1491.11	991.86–2241.66
96	25.2	4.51–35.75	13.15	NA	1189.83	955.19–1482.13	526.19	307.06–901.72

**Table:** (b)

Time (hour)	LC_50 _ (*μ*g/L) for Ni	95% Confidence limits	LC_50 _ (*μ*g/L) for Fe	95% Confidence limits	LC_50 _ (*μ*g/L) for Al	95% Confidence limits	LC_50 _ (*μ*g/L) for Mn	95% Confidence limits
24	57280.92	49144.72–66764.11	911.44	654.2–1200.59	12530.33	10197.69–15396.55	3984.34	2341.04–16025.68
48	39177.81	33011.57–46495.85	644.39	450.21–770.2	6980.05	2360.23–20642.53	1733.27	1050.82–3095.45
72	35134.49	28902.18–42710.69	521.24	300.04–617.27	4964.23	3598.66–6848.01	636.03	278.65–943.71
96	19743.75	14774.15–26384.97	278.9	NA	3101.96	1281.61–7507.85	510.24	206.07–689.26

NA: not available: values could not be calculated from probit software.

**Table 3:** Comparison of LC_50_ (or EC_50_) values of freshwater ostracod *S. major* with other freshwater crustacean (ostracod, cladoceran, and amphipod).

Metal	Species	Live stage	Test duration	LC_50_ (*μ*g/L)	Reference
Copper	*Hyalella azteca *	Adult	96 h	912	[20]
	*Daphnia magna*	24 h	48 h	73.1	[18]
	*Gammarus fasciatus*	Adult	48 h	190	[21]
	*Daphnia carinata*	6 hours	96 h	41	[19]
	*Cypris subglobosa*	Adult	48 h	550*	[10]
	*S. major*	Adult	96 h	25.2	This study

Cadmium	*Hyalella azteca*	Adult	96 h	17.5	[20]
	*Echinogammarus meridionalis*	Adult	96 h	36.17	[22]
	*Daphnia magna*		96 h	12.7	[23]
	*Gammarus pulex*	Adult	96 h	82.1	[24]
	*Cypris subglobosa*	Adult	48 h	821*	[10]
	*S. major*	Adult	96 h	13.1	This study

Zinc	*Hyalella azteca*	Adult	96 h	1613	[20]
	*Daphnia magna*	24h	48 h	752.8	[18]
	*Echinogammarus meridionalis*	Adult	96 h	4610	[22]
	*Cypris subglobosa*	Adult	24 h	3400*	[10]
	*S. major*	Adult	96 h	1189	This study

Lead	*Hyalella azteca*	Adult	96 h	18	[25]
	*Daphnia magna*	24 h	48 h	55641	[18]
	*Daphnia carinata*	Neonate	48 h	170	[19]
	*Cypris subglobosa*	Adult	48 h	40190*	[10]
	*S. major *	Adult	96 h	526	This study

Nickel	*Cypris subglobosa*	Adult	48h	75780*	[10]
	*Daphnia magna*	Adult	48 h	7290*	[26]
	*S. major*	Adult	96 h	19743	This study

Iron	*Cypris subglobosa*	Adult	48 h	115200*	[10]
	*Daphnia magna*	Adult	48 h	7200*	[26]
	*Ceriodaphnia dubia*	<24 h	48 h	36690	[27]
	*Daphnia pulex*	neonate	48 h	12930	[28]
	*S. major*	Adult	96 h	278	This study

Aluminium	*Cypris subglobosa*	Adult	48 h	100900	[10]
	*Daphnia magna*	Adult	48 h	32000*	[26]
	*S. major*	Adult	96 h	3101	This study

Manganese	*Cypris subglobosa*	Adult	48 h	11770	[10]
	*Daphnia magna*	Adult	48 h	8280*	[26]
	*S. major*	Adult	96 h	510	This study

*EC_50_ value (immobilization).
